# Estradiol promotes cells invasion by activating β-catenin signaling pathway in endometriosis

**DOI:** 10.1530/REP-15-0371

**Published:** 2015-12

**Authors:** Wenqian Xiong, Ling Zhang, Lan Yu, Wei Xie, Yicun Man, Yao Xiong, Hengwei Liu, Yi Liu

**Affiliations:** Department of Obstetrics and Gynecology, Tongji Medical College, Union Hospital, Huazhong University of Science and Technology, Wuhan, 430022, China; Department of Obstetrics and Gynecology, Union Hospital, Huazhong University of Science and Technology, 1277 JieFang Road, Wuhan, China

## Abstract

Endometriosis is an estrogen-dependent disease that involves the adhesion, invasion, and angiogenesis of endometrial tissues outside of the uterine cavity. We hypothesized that a link exists between estrogen and beta-catenin (β-catenin) signaling in the pathogenesis of endometriosis. Human endometrial stromal cells (HESCs) were separated from eutopic endometrial tissues that were obtained from patients with endometriosis. β-catenin expression and cells invasiveness ability were up-regulated by 17β-estradiol (E_2_) in an estrogen receptor (ESR)-dependent manner, whereas β-catenin siRNA abrogated this phenomenon. Moreover, co-immunoprecipitation and dual immunofluorescence studies confirmed ESR1, β-catenin, and lymphoid enhancer factor 1/T cell factor 3 co-localization in the nucleus in HESCs after E_2_ treatment. To determine the role of β-catenin signaling in the implantation of ectopic endometrium, we xenotransplanted eutopic endometrium from endometriosis patients into ovariectomized severe combined immunodeficiency mice. The implantation of the endometrium was suppressed by β-catenin siRNA. Collectively, studies regarding β-catenin signaling are critical for improving our understanding of the pathogenesis of estrogen-induced endometriosis, which can translate into the development of treatments and therapeutic strategies for endometriosis.

## Introduction

Endometriosis is a common benign gynecological disease that is defined as the presence of endometrial tissue outside the uterine cavity. This disease affects women of reproductive age and causes pelvic pain and infertility. The incidence rate of endometriosis is ∼10% (Giudice & Kao 2004). The pathogenesis of endometriosis is multifactorial, and endogenous and exogenous estrogens play key roles in its development and progression (Huhtinen *et al*. 2012). Studies have shown greater synthesis of 17β-estradiol (E_2_) in ectopic endometrium than in eutopic endometrium, which is critical for endometrial implantation (Delvoux *et al*. 2009). Accordingly, the inhibition of systemic estrogen action is the current medical therapy for endometriosis and results in restricted proliferation. Aberrant production of estrogen plays an indispensable role in the pathogenesis of this disease.

Beta-catenin (β-catenin) acts as both a regulator of cell adhesion and migration in its cadherin-bound form and a transcriptional factor in Wnt signaling that plays a key role in the regulation of proliferation, growth, and differentiation in endometrial physiology and disease (van der Horst *et al*. 2012). Wnts binding to Frizzled results in the activation of receptor kinases, phosphorylation of the cytoplasmic mediator Dishevelled, and inhibition of the multifunctional serine/threonine kinase glycogen synthase kinase 3, ultimately allowing for the accumulation of β-catenin (Mulholland *et al*. 2005). β-catenin then translocates into the nucleus and associates with transcription factors of the T cell factor/lymphoid enhancer factor (TCF/LEF) family to induce the expression of its downstream target genes (Akiyama 2000, Peifer & Polakis 2000). A previous study of Matsuzaki *et al*. (2010) suggested that aberrant β-catenin expression is found in the endometrium of patients with endometriosis. Moreover, endometriotic lesion development was shown to be inhibited by targeting the β-catenin/TCF complex (Matsuzaki & Darcha 2013). Many *in vivo* physiological studies regarding the cross-talk between estrogen and β-catenin signaling have been conducted in tissues of the brain (Cardona-Gomez *et al*. 2004) and uterus (Gunin *et al*. 2004). In neuroblastoma cells, β-catenin is stabilized by estrogen in neurons, exerting a significant effect at the transcriptional level (Varea *et al*. 2009). The growing number of nuclear receptors interacting with β-catenin causes alterations in cell proliferation and tumorigenesis (Mulholland *et al*. 2005). Based on the results of the cited studies, we hypothesized that a link exists between estrogen and β-catenin signaling in the pathogenesis of endometriosis.

The aims of the present study were to investigate the molecular mechanism underlying the interaction between estrogen and β-catenin signaling in human endometrial stromal cells (HESCs), illustrate whether the β-catenin signaling pathway plays a critical role in estrogen-facilitated invasion and angiogenesis by HESCs, and determine the role of β-catenin in a non-obese diabetic severe combined immunodeficiency (NOD–SCID) mouse endometriosis model.

## Materials and methods

### Tissue collection

All eutopic endometrial tissues were obtained from endometriosis patients by hysterectomy, which was performed at the Department of Obstetrics and Gynecology, Tongji Medical College, Union Hospital, Huazhong University of Science and Technology. The average age was 39 years (range 33–44) at the time of laparoscopy. The patients had not received any hormonal treatment for at least 6 months before the surgical procedure. Informed consent was obtained from each patient using protocols approved by the Human Investigation Committee at Tongji Medical College, Union Hospital, Huazhong University of Science and Technology. All samples were obtained in the proliferative phase of the cycle, which was confirmed histologically according to established criteria.

### Cell culture

Isolation and culture of HESCs began with the collection of eutopic tissues under sterile conditions. The tissues were then transported to the laboratory on ice in a 1:1 formula of DMEM/F12 (Gibco) with 10% fetal bovine serum (FBS; Gibco). The minced eutopic endometrium was digested with collagenase type 2 (0.1%; Sigma–Aldrich) for 30 min at 37 °C with constant agitation. The tissue pieces were filtered on a 100 μm wire sieve to remove debris. Following gentle centrifugation, the supernatant was discarded, and the cells were resuspended in DMEM/F12. The HESCs were separated from epithelial cells by passing them over a 400 μm wire sieve. The filtered suspension was layered over Ficoll and centrifuged at 1200 ***g*** for 20 min to further remove leukocytes and erythrocytes, and the middle layer was collected and then washed with D-Hanks solution. The HESCs were placed in a culture flask and allowed to adhere for 24 h. The adherent stromal cells were cultured as a monolayer in flasks with DMEM/F12 containing 15% FBS, 20 mmol/l HEPES, 100 IU/ml penicillin, and 100 μg/ml streptomycin and incubated in 5% CO_2_ at 37 °C. This method supplied 95% pure HESCs.

### Hormone treatment

E_2_ was purchased from Sigma–Aldrich (E-2758) and dissolved in DMSO. ICI 182 780 (ICI), an estrogen receptor antagonist was purchased from Cayman Chemical (CAS 129453-61-8; Ann Arbor, MI, USA) and dissolved in DMSO. Cells were incubated with E_2_ at various doses for various times or with DMSO (control group).

### RT-PCR

Total RNA was extracted from cultured cells using TRIzol reagent (Invitrogen Life Technologies), and 1 μg RNA was used for cDNA synthesis. SYBR Premix Ex Taq II (#RR820A, Takara, Dalian, China) was used for PCR. Each reaction mixture consisted 5 μl 2× SYBR Premix Ex Taq II, 0.2 μM forward PCR primer, 0.2 μM reverse PCR primer, 0.2 μl 50× ROX reference dye, 1 μl cDNA, and 3.4 μl sterile distilled water in a final volume of 10 μl. Quantitative real-time PCR was performed using the preset PCR program of the StepOnePlus Real-Time PCR System (Applied Biosystems, Inc.) to quantify mRNA expression, with β-actin as an internal control. Primers for quantitative RT-PCR analyses were synthesized based on the GenBank database. The primers were as follows: β-catenin sense, 5′-AGCT TCCA GACA CGCT ATCA T-3′; β-catenin antisense, 5′-CGGT ACAA CGAG CTGT TTCT AC-3′; matrix metalloproteinase 9 (MMP9) sense, 5′-TGTA CCGC TATG GTTA CACT CG-3′; MMP9 antisense, 5′-GGCA GGGA CAGT TGCT TCT-3′; vascular endothelial growth factor (VEGF) sense, 5′-GAGG AGCA GTTA CGGT CTGT G-3′; VEGF antisense, 5′-TCCT TTCC TTAG CTGA CACT TGT-3′. The following PCR conditions were used: 95 °C for 10 s; 35 cycles of 95 °C for 5 s, 60 °C for 30 s, and 72 °C for 30 s and a dissociation program of 95 °C for 15 s, 60 °C for 30 s, and 95 °C for 15 s. The expression levels of the target genes were calculated from the ΔΔ*C*t values.

### Western blot analysis

After the cells were washed with PBS, cell extracts were prepared in lysis buffer containing 40 mM Tris–HCl, 100 mM NaCl, and 0.1% Nonidet P-40 and supplemented with protease inhibitors. Whole-cell lysates were collected by centrifugation at 16000 ***g*** at 24 °C for 10 min after the cells were incubated for 30 min on ice. Cytoplasmic and nuclear proteins were extracted according to the instructions of a cytoplasmic and nuclear protein extraction kit (Sangon Biotech, Shanghai, China). Cells were harvested in ice-cold PBS, scraped from culture dishes on ice using a plastic cell scraper, and collected in 1.5 ml micro-centrifuge tubes. The cytoplasmic lysis buffer contains 1 ml hypotonic buffer, 5 μl phosphatase inhibitor, 1 μl dl-dithiothreitol (DTT), and 10 μl phenylmethanesulfonyl fluoride (PMSF). The material was centrifuged at 3×1000 r.p.m. for 5 min to sediment the nuclei. The supernatant was then centrifuged at 12×1000 r.p.m. for 10 min to get the non-nuclear supernatant fraction. The nuclear pellet was then washed three times with cytoplasmic lysis buffer and resuspended in nuclear lysis buffer (1 ml lysis buffer, 5 μl phosphatase inhibitor, 1 μl DTT, and 10 μl PMSF). The mixture was sonicated briefly to aid nuclear lysis. The nuclear lysates were collected after centrifugation at 12×1000 r.p.m. for 10 min at 4 °C. The samples were boiled for 10 min and then separated by 10% SDS–PAGE. The proteins were transferred to a PVDF membrane (Millipore, Billerica, MA, USA) in the presence of 20% methanol and 0.1% SDS. Nonspecific signals were blocked by incubating the membrane in 5% nonfat milk in Tris-buffered saline–Tween-20 for 1 h. The membranes were incubated with primary antibodies of interest overnight at 4 °C. The antibodies were directed against the following proteins: β-catenin (#8480, Cell Signaling Technology, Danvers, MA, USA), non-phospho β-catenin (#8814, Cell Signaling Technology), GAPDH (ab181602, Abcam, Cambridge, UK), or histone H1 (ab61177, Abcam). The blots were developed using HRP-conjugated secondary antibodies (Cell Signaling Technology), and the proteins were visualized by an ECL procedure (Millipore) according to the manufacturer's recommendations.

### siRNAs and transfection

HESCs were grown in culture medium with 15% FBS before transfection. When the cells were 50–60% confluent, β-catenin siRNA (RiboBio, Guangzhou, China) and Lipofectamine 2000 (Invitrogen) were added to opti-MEM, mixed, incubated for 20 min, and then added to the cells at room temperature (RT) according to the manufacturer's protocol. Scrambled siRNA (RiboBio) was used as a control. After 6 h, the mixture was replaced with phenol red-free DMEM/F12 with 15% FBS in 5% CO_2_ at 37 °C.

### Plasmids and dual-luciferase assay

TOPflash, a TCF reporter plasmid; FOPflash, a negative control for TOPflash; and pRL-SV40, a *Renilla* luciferase expression plasmid, were purchased from GeneChem (Shanghai, China). Co-transfection experiments were performed in 24-well plates. In total, 1×10^5^ cells were seeded per well in 500 μl medium. Then, 0.8 μg TOPflash or FOPflash and 0.008 μg pRL-SV40 reporter plasmid plus Lipofectamine 2000 transfection reagent were added to the HESCs. After 48 h, luciferase activity was measured using the Dual-Luciferase Reporter Assay System (Promega). Then, firefly luciferase activity was normalized to the corresponding *Renilla* luciferase activity.

### Matrigel invasion assay

Transwell units with 8.0 μm pore-size polycarbonate filters (Corning Costar, Tewksbury, MA, USA) were precoated with 50 μl 1:2 diluted Matrigel (Sigma–Aldrich) and used to investigate cell invasion. A total volume of 100 μl suspension containing ∼10^5^ cells/ml was added to each upper compartment of the precoated units. After the cells were allowed to attach for 30 min, the units were transferred to wells containing 500 μl DMEM/F12 medium with 20% FBS as a chemoattractant and incubation was conducted for 24 h. After removing the cells and Matrigel from the upper surface of the membrane with a cotton bud and staining with 0.1% crystal violet for 15 min, the number of cells on the underside was determined using light microscopy (Olympus, Japan). Five randomly selected fields were counted per insert.

### Co-immunoprecipitation

For co-immunoprecipitation (COIP) assays, cells were lysed in 400 μl lysis buffer (20 mM Tris (pH 7.4), 50 mM NaCl, 1 mM EDTA, 0.5% NP-40, 0.5% SDS, 0.5% deoxycholate, and protease inhibitors). Lysate aliquots (500 μg at 1 μg/μl) were precleared with 60 μl protein A-sepharose beads (Beyotime, Haimen, China) for 1 h at 4 °C. Appropriate amounts of mouse-directed antibody against β-catenin (sc-133239, Santa Cruz Biotechnology), mouse antibody directed against estrogen receptor alpha (ESR1; sc-73479, Santa Cruz Biotechnology), and mouse nonspecific IgG (Biosense, Bergen, Norway) were then added and incubated for 4 h at 4 °C. Preblocked agarose beads (100 μl) were added to the antibody/lysate mixture and incubated overnight at 4 °C. After the beads were washed three times with 1× PBS, the bound proteins were eluted in SDS sample buffer, resolved by SDS–PAGE, and analyzed by immunoblotting. The antibodies used for immunoblotting were rabbit directed antibodies against ESR1 (ab75635, Abcam), β-catenin (#8814, Cell Signaling Technology), LEF1 (#2286, Cell Signaling Technology), and TCF3 (#2883, Cell Signaling Technology).

### Dual immunofluorescence

HESCs were cultured in 25 mm^2^ dishes and treated with E_2_ (Sigma–Aldrich) at 10^−8^ mol/l for 48 h, with DMSO as a control. Then, the cells were fixed in ice-cold 4% paraformaldehyde for 10 min, washed with 1× PBS, and permeabilized in PBS containing 0.5% Triton X-100 for 3 min at RT. All cells were blocked (5% BSA, 30 min, RT) and incubated overnight (4 °C) with primary antibodies directed against β-catenin (#8814, Cell Signaling Technology), LEF1 (#2286, Cell Signaling Technology), TCF3 (#2883, Cell Signaling Technology), and ESR1 (ab75635, Abcam). After the cells were exposed to the primary antibodies, they were incubated with Alexa Fluor 488- and 568-conjugated secondary antibodies and mounted with Prolong Gold antifade medium. Immunofluorescently labeled cell monolayers were visualized under an Olympus FV1000 laser scanning confocal microscope (Olympus).

### Xenotransplantation of human eutopic endometrial tissues

Proliferative-phase eutopic endometrial tissues were obtained from ten premenopausal women with endometriosis during hysterectomy at Wuhan Union Hospital. Fresh endometrial tissue samples were fragmented into 2–3 mm^3^ sections under sterile conditions. The fragments were cultured in phenol red-free DMEM/F12 (1:1)+10% FBS before transplantation into NOD–SCID mice (Pearson *et al*. 2008). In total, 50 8-week-old NOD–SCID female mice were used. The guidelines for animal care were approved by the Institutional Animal Care and Use Committee, Tongji Medical College, Huazhong University of Science and Technology. The mice were bilaterally ovariectomized and then left untreated for 14 days. Eight to ten fragments of human endometrial lesions were implanted into the pelvic and peritoneal cavity (Grummer *et al*. 2001). The mice were divided into six groups at 2 days after implantation. Mice were co-treated with E_2_ (0.2 μg/day) plus positive β-catenin siRNA (125 μg/kg per day) or scrambled siRNA (125 μg/kg per day) and saline solution control. All treatments were administrated via peritoneal injection. E_2_ was purchased from Sigma–Aldrich (Y0000852). Mice were sacrificed 10 and 21 days after implantation, the peritoneal cavity was examined, and tissue collected. The implanted endometrial lesions were harvested for hematoxylin and eosin staining and for immunohistochemical (IHC) analysis. Western blot analysis of the tissues was performed as described previously.

### Statistical analysis

All values are shown as the mean±s.e.m. Unpaired *t*-tests and one-way ANOVA were used to demonstrate statistical significance. Statistical significance was set at *P*<0.05. All experiments were performed in triplicate.

## Results

### E_2_ up-regulates β-catenin mRNA and protein expression with the involvement of ERs in HESCs

**Figure 1:**
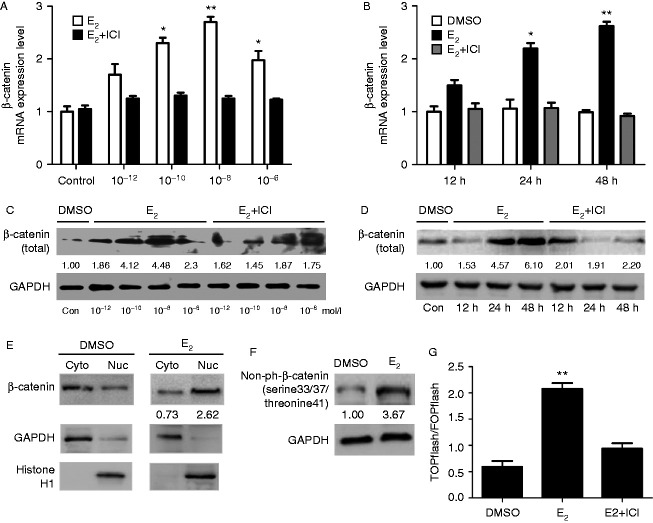
(A, B, C and D) Effects of E_2_ and E_2_+ICI on the β-catenin expression. (A and C) Dose-dependence by the stimulation with E_2_. HESCs were cultured in a phenol red-free DMEM/F12 for 24 h and incubated with various concentrations of E_2_ and E_2_+ICI for 24 h. (B and D) Time course by the stimulation with E_2_. Cells were cultured in a phenol red-free DMEM/F12 for 24 h and treated with E_2_ (10^−8^ mol/l), E_2_+ICI for the indicated times (0, 12, 24, and 48 h). β-catenin mRNA was extracted by TRIzol and examined by RT-PCR (A and B). Protein samples were separated by 10% SDS–PAGE and subjected to western blot analysis for total β-catenin (C and D). (E) Effects of E_2_ on the nuclear localization of β-catenin in HESCs. Cells were stimulated with DMSO or E_2_ (10^−8^ mol/l) for 48 h, followed by western blot, and the cytoplasmic and nucleus of β-catenin was observed. (F) Effects of E_2_ on the non-phosphorylated β-catenin (active β-catenin) in HESCs. Cells were stimulated with DMSO or E_2_ (10^−8^ mol/l) for 48 h, followed by western blot, and non-phosphorylated β-catenin was observed. (G) E_2_ increases TCF transcriptional activity. TOPflash or FOPflash was co-transfected with pRL-SV40 into HESCs. After transfection for 24 h, the cells were stimulated with E_2_ (10^−8^ mol/l) and E_2_ plus ICI for 48 h. The luciferase activities are shown as percentages of the control level. Data are expressed as mean±s.e.m. *P<0.05 and **P<0.01 vs controls. Data presented are from three independent experiments.

Endometriosis is an estrogen-dependent disease (Huhtinen *et al*. 2012). Abnormal β-catenin protein expression has been identified in ovarian endometrioid adenocarcinoma associated with endometriosis (Stewart *et al*. 2013), and E_2_ has been shown to stabilize β-catenin expression through estrogen receptor binding in neuronal cells (Varea *et al*. 2009). First, we tested the relationship between E_2_ and β-catenin, a key transcription factor in Wnt signaling. The qRT-PCR results showed that E_2_ was capable of inducing β-catenin expression in a dose- and time-dependent manner. E_2_ caused up-regulation of β-catenin mRNA at 10^−10^ mol/l (∼2.3-fold), followed by maximal up-regulation at 10^−8^ mol/l (∼2.8-fold) and a decline at 10^−6^ mol/l (∼1.9-fold) while remaining high compared with the control (Fig. 1A). As shown in Fig. 1B, qRT-PCR analysis demonstrated that E_2_ (10^−8^ mol/l) treatment caused β-catenin up-regulation (∼2.2-fold) at 24 h and that this up-regulation continued to increase until 48 h (∼2.9-fold). Next, we explored whether this E_2_-regulated response was reflected in the protein level of β-catenin. Western blot analysis demonstrated that β-catenin was up-regulated (∼4.1-fold) at 10^−10^ mol/l E_2_, then increased (∼4.5-fold) at 10^−8^ mol/l E_2_ but slightly declined at 10^−6^ mol/l E_2_ (Fig. 1C). β-catenin protein expression was also up-regulated (∼4.6-fold) at 24 h and continued to increase until 48 h (∼6.1-fold) at 10^−8^ mol/l E_2_ (Fig. 1D). E_2_ up-regulated β-catenin mRNA and protein expression in a dose- and time-dependent manner in HESCs, and the effect was maximal at 10^−8^ mol/l E_2_ at 48 h (**P<0.01). Furthermore, we wanted to determine whether the estrogen-regulated responses are dependent on estrogen receptor (ESR). To address this question, HESCs were treated with ICI (ER inhibitor, 10^−6^ mol/l) in conjunction with E_2_ at various doses and times. We observed that the E_2_-regulated inductive responses of β-catenin mRNA and protein were indeed antagonized by ICI (Fig. 1A, B, C and D). Collectively, these suggested that estrogen induces β-catenin up-regulation in HESCs via the involvement of ER.

### E_2_ activates the β-catenin signaling pathway in HESCs

Next, we wanted to confirm the effects of E_2_ on the β-catenin signaling pathway in HESCs. To examine whether E_2_ alters the subcellular distribution of β-catenin, cells were treated with E_2_ (10^−8^ mol/l) or with control (DMSO) for 48 h. Western blot analysis indicated that E_2_ treatment led to an increase in β-catenin protein levels in the nucleus (∼2.6-fold) and to a slight decrease in β-catenin protein levels in the cytoplasm compared with the control (∼0.7-fold) (Fig. 1E). As shown in Fig. 1F, non-phosphorylated β-catenin (active β-catenin) was up-regulated by E_2_ (∼3.67-fold) compared with the control. Next, we investigated the effect of E_2_ on TCF-induced transcriptional activity in HESCs. As shown in Fig. 1G, TOPflash activity markedly increased (**P<0.01) upon stimulation with E_2_ and could be abolished by ICI (**P<0.01). Collectively, these findings demonstrated that E_2_ activates β-catenin signaling in HESCs.

### E_2_ stimulates ESR1 and β-catenin co-localization in the nucleus in HESCs

**Figure 2:**
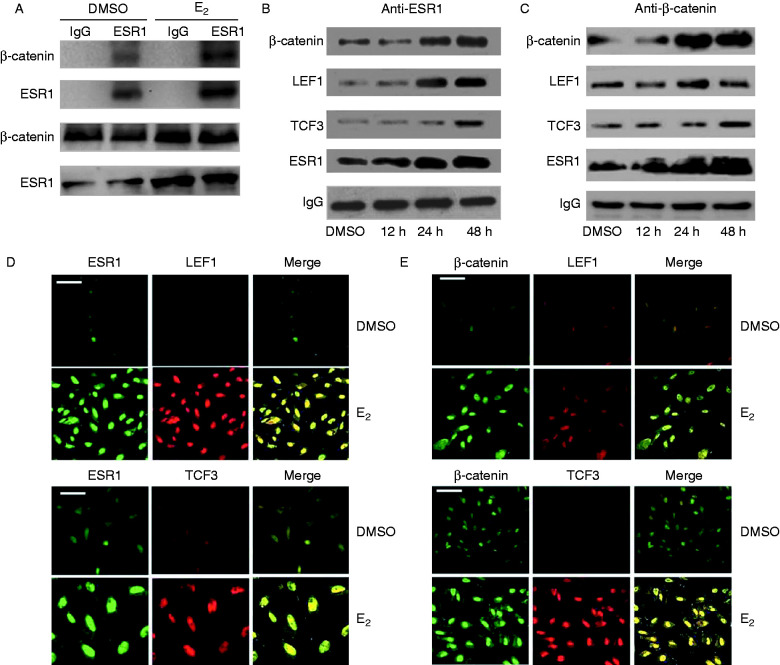
(A) The interaction between ESR1 and β-catenin in HESCs was examined. Cells were incubated with DMSO and E_2_ (10^−8^ mol/l) for 24 h. Then cell lysates were prepared and immunoprecipitated with IgG and ESR1 antibody. The second panel is ESR1 shown as an internal loading control. Immunoblots of input lysate controls (5% of input) are also shown. (B and C) HESCs were incubated with E_2_ (10^−8^ mol/l) under different time scales (0, 12, 24, and 48 h). (B) Cell lysates were prepared and immunoprecipitated with ESR1 antibody or IgG. The interaction between ESR1 and β-catenin/TCF3/LEF1 was examined. (C) Cell lysates were prepared and immunoprecipitated with β-catenin antibody or IgG. The interaction between β-catenin and ESR1/TCF3/LEF1 was examined. (D and E) Co-localization of ESR1, β-catenin, and LEF1/TCF3 in nucleus were stimulated with E_2_ in HESCs. Cells were cultured in phenol red-free DMEM/F12 for 24 h and incubated with DMSO and E_2_ (10^−8^ mol/l) for 48 h. (D) Representative confocal microscopy images of HESCs immunostained for ERS1 (green, left panels) and its co-localization with the LEF1 and TCF3 (red, middle panels), and the merged images (right panels). (E) Representative confocal microscopy images of HESCs immunostained for β-catenin (green, left panels) and its co-localization with the LEF1 and TCF3 (red, middle panels), and the merged images (right panels). The bars indicate 20 μm. Data presented are from three independent experiments.

Estrogen induces cellular effects via two molecular mechanisms: a classic genomic mechanism in which E_2_ binds ER to stimulate target gene expression and a nongenomic mechanism in which E_2_ induces cellular effects independent of ER-related transcriptional activity (Acconcia & Kumar 2006). Therefore, we wanted to clarify which mechanism is responsible for the observed effects in HESCs. Further investigation was conducted using COIP. As shown in Fig. 2A, the interaction between ESR1 and β-catenin was significantly more frequent when cells were treated with E_2_. Moreover, we wanted to examine whether TCF3/LEF1 cross-talk with ESR1. As shown in Fig. 2B, the interaction between ESR1 and β-catenin/TCF3/LEF1 were detected at all time points (0, 12, 24, and 48 h) by E_2_ (10^−8^ mol/l) treatment, and the effect was maximal at 48 h. E_2_ stimulated ESR1 binding to β-catenin/TCF3/LEF1 in a time-dependent manner. Furthermore, β-catenin pulldown analysis detected an ESR1/TCF3/LEF1 complex. In contrast, the effects of ESR1, TCF3, and LEF1 interactions with β-catenin were maximal at 24 h (Fig. 2C). Dual immunofluorescence studies revealed intense nuclear co-localization, with ESR1, β-catenin, and LEF1/TCF3 co-localizing in the nucleus in HESCs after treatment with E_2_ for 48 h. The expression of these proteins in the control (DMSO) group also showed a co-localization pattern, but the co-localization was less intense than that in the E_2_ group (Fig. 2D and E). These results suggested that E_2_ induced β-catenin activation and led to a molecular association with ESR1 and LEF1/TCF3 in HESCs.

### β-catenin deletion represses E_2_-induced expression of MMP9 and VEGF in HESCs

**Figure 3:**
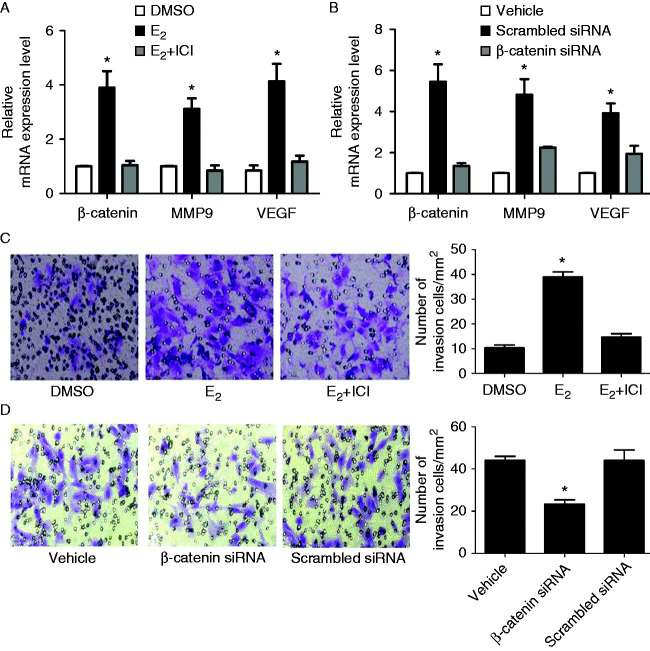
(A) Effect of E_2_ on VEGF and MMP9 expression. Cells were stimulated with vehicle, E_2_ (10^−8^ mol/l), and E_2_ (10^−8^ mol/l)+ICI for 48 h. β-catenin, MMP9, and VEGF mRNA were detected by qRT-PCR. (B) Effect of β-catenin siRNA on VEGF and MMP9 expression. After β-catenin, siRNA was transfected for 24 h, the cells were stimulated with vehicle and E_2_ (10^−8^ mol/l) for 48 h. β-catenin, MMP9, and VEGF were detected by qRT-PCR. (C) E_2_ enhances the invasive ability of HESCs. Left: representative photomicrographs of invasion of vehicle, E_2_, and E_2_+ICI-treated HESCs. Right: number of invasive cells/mm^2^ in DMSO, E_2_, and E_2_+ICI-treated HESCs. (D) Effect of β-catenin deletion on cells invasiveness ability stimulation with E_2_. Left: representative photomicrographs of invasion of control, β-catenin siRNA, or scrambled siRNA transfected HESCs. Right: number of invasive cells/mm^2^ in control, β-catenin siRNA, or scrambled siRNA transfected HESCs. Data are expressed as mean±s.e.m. *P<0.05 vs controls. Data presented are from three independent experiments.

In endometrial implants, VEGF induces neovascularization (Harada *et al*. 2001), and MMPs promote matrix remodeling (Sillem *et al*. 2001). A previous study suggested that MMP levels were significantly higher in epithelial and stromal cells in patients with endometriosis compared to patients without endometriosis (Becker *et al*. 2010). Estrogen has been shown to induce VEGF and MMP9 production in endometriotic lesions, leading to the adhesion and growth of endometrial cells, which can spill into the peritoneal cavity (Wang *et al*. 2011). These two factors seem to be vital for the establishment of endometriosis. Therefore, we focused on whether VEGF and MMP9 are up-regulated by E_2_ interaction with the β-catenin signaling pathway. QRT-PCR analysis indicated that E_2_ treatment led to increased levels of MMP9 (∼3.5-fold) and VEGF (∼4.2-fold) in HESCs, whereas ICI treatment significantly abrogated this phenomenon (*P<0.05; Fig. 3A). HESCs were transiently transfected with β-catenin siRNA. This RNA interference resulted in ∼90% knockdown of β-catenin protein levels in the HESCs. The expression levels of VEGF and MMP9 in the transfected HESCs decreased significantly when the cells were also treated with E_2_ (*P<0.05; Fig. 3B). These results suggested that up-regulation of MMP9 and VEGF by E_2_ was dependent on β-catenin.

### β-catenin deletion abrogates the E_2_-induced increase of HESCs invasiveness

After HESCs were treated with E_2_ for 48 h, the Transwell assay revealed that these cells were more invasive than untreated cells or cells treated with E_2_ plus ICI (*P<0.05; Fig. 3C). As shown in Fig. 3D, the Transwell assay revealed that the cells treated with β-catenin siRNA were less invasive than the untreated cells (vehicle) and the negative controls (treated with scrambled siRNA) (*P<0.05). All experimental groups were treated with E_2_ (10^−8^ mol/l) after transfection with siRNA. These data indicated that the E_2_-induced increase in the invasiveness of HESCs was dependent on β-catenin.

### Silencing of the β-catenin gene by siRNA inhibits the adhesiveness and invasiveness of eutopic endometrium induced by E_2_ in a NOD–SCID mouse endometriosis model

**Figure 4:**
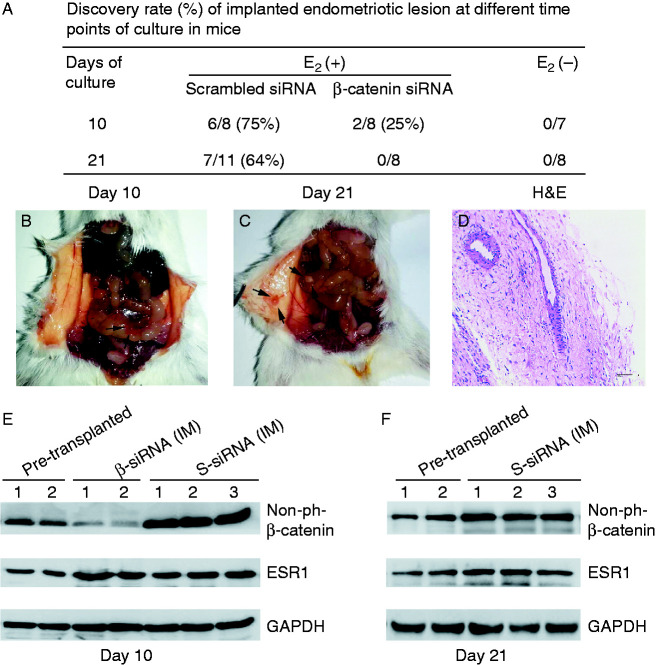
The flow chart and representative pictures of the endometriotic animal model. (A) Fifty NOD–SCID female mice transplanted with human normal endometrium were separated into six groups: mice treated with E_2_ plus positive β-catenin siRNA or scrambled siRNA and saline solution control after a 10-day incubation; mice treated with E_2_ plus positive β-catenin siRNA or scrambled siRNA and saline solution control after a 21-day incubation. (B and C) Representative pictures of the implanted fragments of endometrial tissues into the peritoneal cavity of the NOD–SCID mice after transplantation of normal endometrium. The arrows indicate the implanted endometrial tissues. (D) The murine lesions were examined by histopathology. (E) Western blotting analysis of non-phosphorylated β-catenin and ESR1 expression of pre-transplanted tissues and endometriotic lesions obtained from mice model (a 10-day incubation). (F) Western blotting analysis of non-phosphorylated β-catenin and ESR1 expression of pre-transplanted tissues and endometriotic lesions obtained from mice model (a 21-day incubation). H&E, hematoxylin and eosin stain.

**Figure 5:**
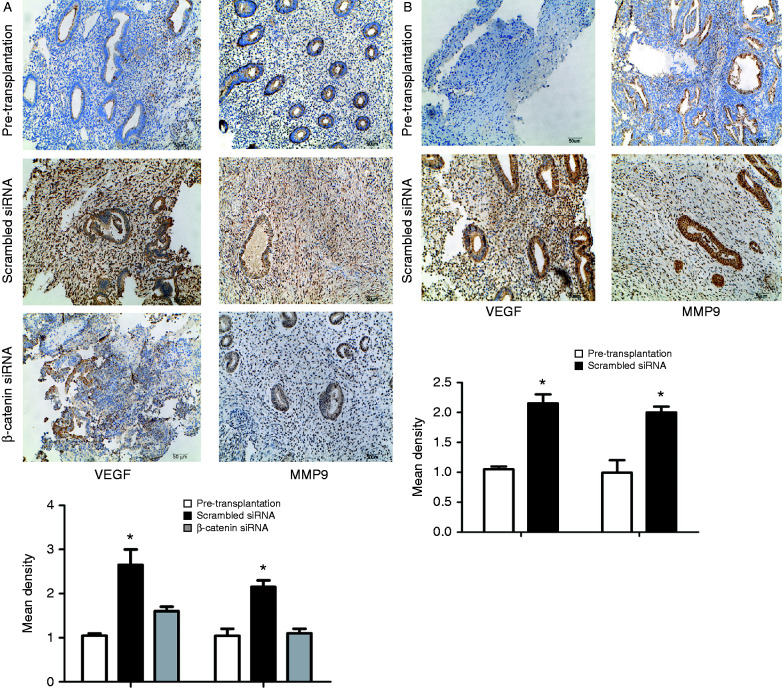
(A) Immunohistochemical (IHC) analysis for VEGF and MMP9 of the samples of pre-transplanted tissues and endometriotic lesions obtained from mice model (a 10-day incubation). (B) IHC analysis for VEGF and MMP9 of the samples of pre-transplanted tissues and endometriotic lesions obtained from mice model (a 21-day incubation). Data are expressed as mean±s.e.m. *P<0.05 vs controls.

To determine whether E_2_ increases the adhesiveness and invasiveness of endometrial lesions through β-catenin signaling *in vivo*, we established an animal model that would mimic ectopic implantation of the endometrium. To generate this model, human eutopic endometrial were implanted into the pelvic and peritoneal cavities of ovariectomized NOD–SCID female mice. Ten days after transplantation, endometriotic lesions were detected in 25% (two of eight) animals treated with E_2_ and scrambled siRNA and 75% (six of eight) of animals treated with E_2_ and β-catenin siRNA (Fig. 4A). Endometriotic lesions were detected in 63.6% (seven of 11 mice) animals treated with E_2_ and scrambled siRNA 21 days after transplantation (Fig. 4A). The discovery rate of mice treated with E_2_ plus scrambled siRNA that developed viable endometriotic lesions was 63.6% (seven of 11 mice) after a 21-day incubation. No visible endometrial fragments were observed in mice treated with E_2_ plus positive β-catenin siRNA after a 21-day incubation or in the control groups after both 10- and 21-day incubations. The lesions were highly vascularized as observed on gross morphological examination (Fig. 4B and C). The murine lesions demonstrated the presence of human endometrial glands and stroma along with epithelial cells lining the lumen by histology (Fig. 4D). To adjust for biological variations, western blot and IHC analyses were performed in samples harvested from the mice during laparotomy and compared with the results for pre-transplantation eutopic endometrium. As shown in Fig. 4E, an increased expression of dephosphorylated β-catenin was demonstrated in the implanted tissues from scrambled siRNA-treated mice compared with their pre-transplantation tissues and with implanted tissues from β-catenin siRNA-treated mice. Implanted tissues from both E_2_ plus positive β-catenin siRNA-treated mice and scrambled siRNA-treated mice displayed up-regulated ESR1 expression compared with their pre-transplantation tissues. Because no visible endometrial fragments were observed in mice treated with E_2_ plus positive β-catenin siRNA after a 21-day incubation, we only used the implanted tissues from scrambled siRNA-treated mice compared with their pre-transplantation tissues. As shown in Fig. 4F, the expression levels of dephosphorylated β-catenin and ESR1 were increased in the implanted tissues from mice treated with E_2_ plus scrambled siRNA. We further performed IHC staining of VEGF and MMP9 in endometriotic lesions. In the 10-day incubation group, increased VEGF (∼2.6-fold) and MMP9 (∼2.4-fold) expression was observed in the endometriotic lesions from mice treated with E_2_ plus scrambled siRNA compared with their pre-transplantation tissues (Fig. 5A). In contrast, the expression of VEGF and MMP9 proteins was attenuated in endometriotic lesions from β-catenin siRNA-treated mice compared to scrambled siRNA-treated mice (Fig. 5A). In the 21-day incubation group, increased MMP9 (∼2.1-fold) and VEGF (∼2.0-fold) expression was demonstrated in the implanted tissues compared with their pre-transplantation counterparts (Fig. 5B). These results confirmed that β-catenin plays a critical role in the E_2_-induced adhesion of implanted endometrial fragments to the peritoneum of mice.

## Discussion

E_2_ is the most potent estrogen found in humans and plays a critical role in the development and progression of endometriosis (Huhtinen *et al*. 2012). The activated β-catenin pathway may interact with multiple nuclear signal transducers to coordinate tissue- or cell-specific functions (Yang *et al*. 2002, Hou *et al*. 2004, Kouzmenko *et al*. 2004, Chandar *et al*. 2005, Armstrong *et al*. 2007). ESR1 and β-catenin have been found to precipitate within the same immune complexes in colon cancer and breast cancer (Kouzmenko *et al*. 2004). The β-catenin signaling pathway also plays a significant role in the estrogen-regulated normal physiological processes of the uterus via a specific protein–protein interaction between β-catenin and ESR1 (Hou *et al*. 2004). However, the cross-talk between E_2_ and β-catenin signaling in the pathogenesis of endometriosis is unknown. β-catenin is a major component of the canonical Wnt signaling pathway. In the absence of the Wnt ligand, this pathway is inactive due to continual degradation of non-junctional β-catenin. In the present study, we found that E_2_ increased β-catenin expression at the mRNA and protein levels in a time- and dose-dependent fashion in HESCs. Furthermore, E_2_ stimulated ESR1 binding to β-catenin. Taken together, our results showed that the β-catenin destruction complex failed to bind to the ESR1/β-catenin conjugation, leading to β-catenin stabilization and eventual translocation to the nucleus. The ESR1/β-catenin conjugation then associated with the transcription factors TCF3 and LEF1. When treated with ICI to antagonize the function of ESR1, β-catenin expression and stabilization decreased.

Adhesion, invasion, and angiogenesis are the steps in the fundamental pathological process of endometriosis. For ectopic implantation and growth to occur, endometrial tissue must first attach itself to the host tissue and then invade the host tissue and obtain its own blood supply from the local vasculature (Giudice *et al*. 1998). VEGF induces neovascularization in endometrial implants (Harada *et al*. 2001). The expression of MMPs in the eutopic endometrium in endometriosis patients differs from that in normal females, and the expression of the MMP9 protein in the endometrium of endometriosis patients is much higher than that in normal females (Collette *et al*. 2006). MMP9 activity is known to be involved in cell invasion (Moon *et al*. 2010, Kang *et al*. 2011). Certain studies have shown that MMP9 and VEGFA are downstream target genes of the Wnt/β-catenin signaling pathway (Wu *et al*. 2007, Qu *et al*. 2014). The present study observed that E_2_ induced an increase in HESCs invasiveness and that ICI blocked this induced increase. β-catenin siRNA was then transfected into HESCs, which demonstrated that invasion was significantly decreased when β-catenin expression was inhibited. Similarly, VEGF and MMP9 gene expression were also inhibited. In the NOD–SCID mouse model, β-catenin siRNA abrogated the implantation of xenotransplanted endometrium mediated by E_2_, suggesting a novel and crucial mechanism in which E_2_ facilitates eutopic endometrial implantation through the β-catenin signaling pathway. However, the mechanism by which ESR1, β-catenin, and the LEF1/TCF3 complex regulate the expression of VEGF and MMP9 is unclear and requires further exploration.

In the present study, we demonstrated that the molecular and functional activation of β-catenin signaling by E_2_ in HESCs leads to the promotion of the nuclear translocation of β-catenin. We further found that ESR1, β-catenin, and LEF1/TCF3 interact in nuclei. All of these results suggested that E_2_ treatment leads to the accumulation of β-catenin in the nucleus, where β-catenin associates with the TCF/LEF family to activate the transcription of downstream target genes, such as the MMP and VEGF genes. These results confirm the importance of the β-catenin signaling pathway in endometriosis under conditions of abnormal estrogen levels.

In summary, the present study demonstrated that E_2_ enhanced β-catenin expression and, as a consequence, β-catenin associated with the LEF/TCF family, increasing HESCs invasion and angiogenesis. In light of the multiple roles of the β-catenin signaling pathway in promoting the development of endometriosis, E_2_ may accelerate disease progression by up-regulating β-catenin expression. Therefore, these findings may provide a potential therapeutic target for the treatment of endometriosis.
